# Generating advanced CAR-based therapy for hematological malignancies in clinical practice: targets to cell sources to combinational strategies

**DOI:** 10.3389/fimmu.2024.1435635

**Published:** 2024-09-20

**Authors:** Shu Zhou, Yuhang Yang, Yulu Jing, Xiaoying Zhu

**Affiliations:** ^1^ Department of Hematology, Zhongnan Hospital, Wuhan University, Wuhan, China; ^2^ The First Clinical Medical College, Wuhan University, Wuhan, China; ^3^ The Second Clinical Medical College, Wuhan University, Wuhan, China; ^4^ National Clinical Research Center for Hematologic Diseases, Jiangsu Institute of Hematology, The First Affiliated Hospital of Soochow University, Suzhou, China; ^5^ Institute of Blood and Marrow Transplantation, Collaborative Innovation Center of Hematology, Soochow University, Suzhou, China

**Keywords:** chimeric antigen receptor T cell, hematological malignancies, targets, allogeneic, combinatorial therapy

## Abstract

Chimeric antigen receptor T (CAR-T) cell therapy has been a milestone breakthrough in the treatment of hematological malignancies, offering an effective therapeutic option for multi-line therapy-refractory patients. So far, abundant CAR-T products have been approved by the United States Food and Drug Administration or China National Medical Products Administration to treat relapsed or refractory hematological malignancies and exhibited unprecedented clinical efficiency. However, there were still several significant unmet needs to be progressed, such as the life-threatening toxicities, the high cost, the labor-intensive manufacturing process and the poor long-term therapeutic efficacy. According to the demands, many researches, relating to notable technical progress and the replenishment of alternative targets or cells, have been performed with promising results. In this review, we will summarize the current research progress in CAR-T eras from the “targets” to “alternative cells”, to “combinational drugs” in preclinical studies and clinical trials.

## Introduction

Immunotherapy, such as adoptive T cell therapy or monoclonal antibody treatment, has long been a major area of interest, and considered to be a promising therapeutic approach in treating hematological or non-hematological malignancies. In recent years, a milestone breakthrough in immunotherapy should be the chimeric antigen receptor T cell (CAR-T) therapy, which utilized T cells genetically modified to express CAR that redirect T-cell specificity towards tumor-associated antigens in an non major histocompatibility complex (MHC) restricted manner (1, 2). The T cell receptor (TCR) was not disrupted and still remained functional on T cells along with the CAR molecule. There were four elements in the CAR molecule including an extracellular antigen binding domain, a hinge region, a transmembrane domain and an intracellular signaling domain. The extracellular domain was usually a single-chain variable fragment (scFv) derived from a Fab or a monoclonal antibody determining the antigen specificity in recognizing tumor cells. The hinge region was the spacer region to expose the antigen binding domain on CAR-T cell surface for binding to target antigens. The transmembrane domain was used to dock CAR in the immune cell membrane. The intracellular domain was composed of the fused signaling domains from the CD3ζ chain of a TCR or FcRγ and costimulatory molecules, such as CD28, 4-1BB, ICOS or OX40, imitating the costimulatory signal of the TCR during activation (3, 4).

As a promising therapeutic regimen, CAR-T cell therapy has stood the test of time for over 30 years, and has developed to the fifth generation. In the first generation of CAR-T which firstly proposed by Eshhar et al., the intracellular region only included the CD3ζ chain as an intracellular stimulation signal, and its activation, proliferation and anti-tumor ability were limited (5). Based on this, the second generation of CAR-T cells had one co-stimulatory domain CD28, 4-1BB, ICOS or OX40 in the intracellular domain, which were significantly enhanced in the activation, proliferation, cytokine secretion and anti-tumor ability (6–8). In order to further enhance the efficacy of CAR-T cells, several researchers proposed the third generation of CAR-T cells which were composed of two costimulatory domains in the intracellular region (9–11). In addition to costimulatory signals like CD28 and (or) 4-1BB, the so-called fourth generation of CAR-T cells were also equipped with a nuclear factor of activated T cell-responsive expression element to overcome the immunosuppressive tumor microenvironment, such as PD1 inhibitor, IL-12 or IL-15 (12–14). Currently, the split, universal, and programmable (SUPRA) CAR system, regarded as the fifth generation of CAR-T cells, were in the spotlight. The SUPRA CAR system was split into two interactive parts, a universal signaling module receptor on T cells and a scFv adaptor molecule to recognize targets, holding promises for the achievement of “off the shelf” cancer immunotherapy with safety, specificity and controllability (15).

**Figure 1:**
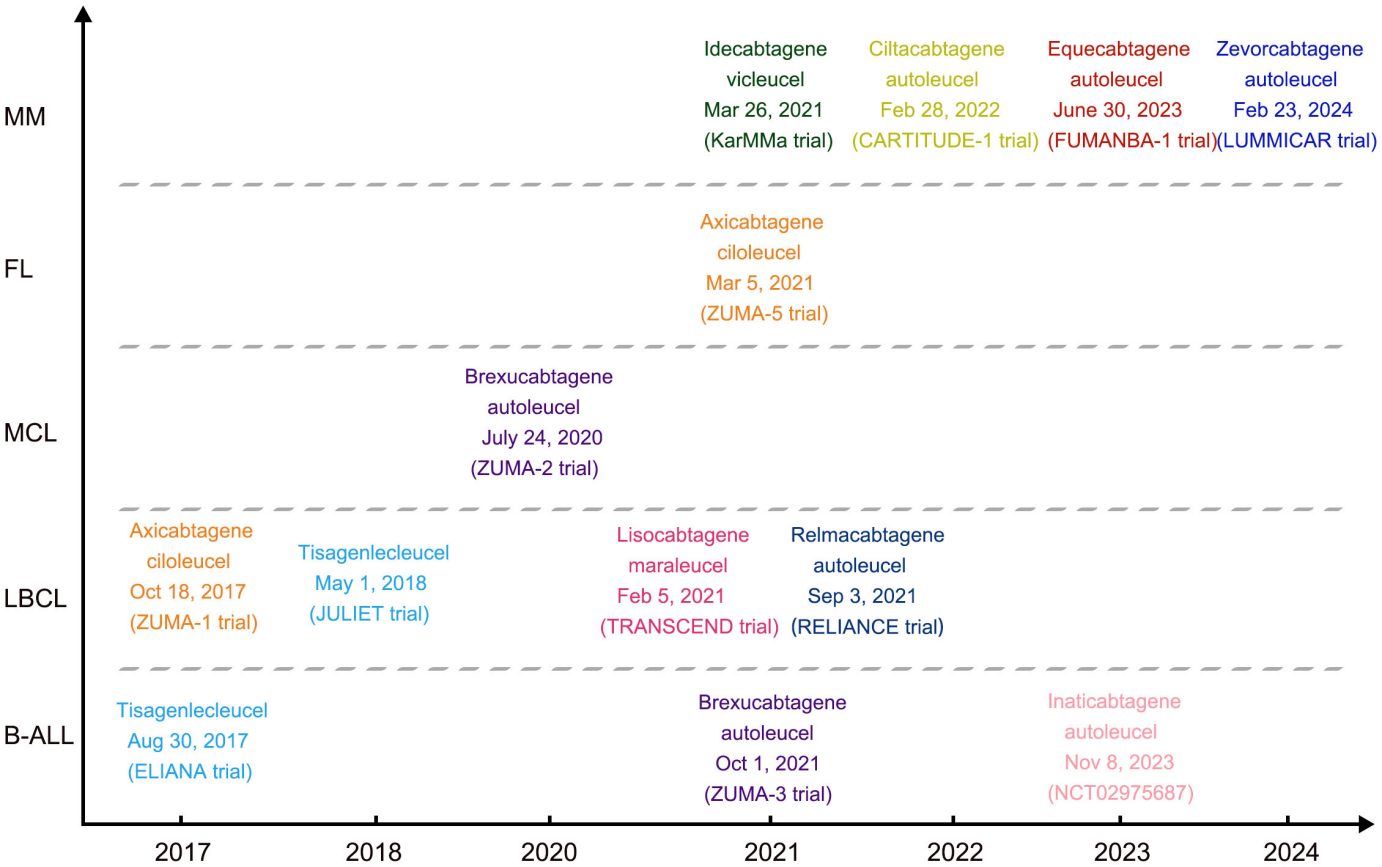
Timeline of FDA or NMPA approved CAR-T products.

As far, six CAR-T products were approved by the United States Food and Drug Administration (FDA) and four were by the China National Medical Products Administration (NMPA) for the treatment of relapsed or refractory (R/R) hematological malignancies (**Figure 1**). However, there were still several limitations to the broad application of CAR-based immunotherapy, such as the lack of ideal targets in acute myeloid leukemia (AML) and T-cell malignancies, the lengthy manufacturing processes, and the poor long-term therapeutic efficacy. In an effort to overcome these obstacles, abundant innovative strategies are currently being investigated. In this review, we will summarize the current research progress in CAR-T eras from the “targets” to “alternative cells” and to “combinational strategies” in clinical practice.

## Targets for CAR-T therapy in hematological malignancies

To construct the CAR-T therapy, the first and most important step was to figure out the ideal targets that could distinguish the malignant cells and normal cells. Although it has not been achieved to identify antigens that were strictly expressed in malignant cells so far, a number of tumor-associated antigens and even antigens that were also highly expressed in normal cells have been discovered and applied for CAR-T therapy. Here, we summarized the ones that were developed under marketing authorization and the others under clinical studies.

### Under marketing authorization

The first FDA-approved CAR-T therapy was tisagenlecleucel, an autologous anti-CD19 CAR-T cell therapy produced by Novartis, which was approved by FDA on August 30, 2017 for the treatment of patients at the age of ≤25 years with B cell precursor acute lymphoblastic leukemia (pre-B ALL) that was refractory or in second or later relapse. Recently, the report of three-year update of “ELIANA Trial” evaluated the favorable long-term safety and effectiveness of tisagenlecleucel. In the trial with a median follow-up of 38.8 months, the overall remission rate (ORR) was 82% with the event-free survival (EFS) rate 44% (95% CI, 31 to 57). The overall survival (OS) was 63% (95% CI, 51 to 73) at 3 years, and most events occurred within the first 2 years. The estimated 3-year relapse-free survival (RFS) with and without censoring for subsequent therapy were 52% (95% CI, 37 to 66) and 48% (95% CI, 34 to 60), respectively. Grade 3/4 adverse events were reported in 29% of patients > 1 year after infusion, and patients reported improvements in quality of life up to 36 months after infusion (16). In addition, tisagenlecleucel was latter approved by FDA on May 1, 2018, for R/R large B-cell lymphoma based on a multi-center JULIET trial. The long-term follow-up analysis of this JULIET trial showed that the ORR was 53.0% in evaluable 115 patients, 45 (39%) patients having a complete remission (CR) as their best overall response at a median follow-up of 40.3 months (17).

Axicabtagene ciloleucel, the second FDA-approved autologous CD19 CAR-T therapy, was applied to large B-cell lymphoma after two or more lines of systemic therapy, including DLBCL, primary mediastinal large B-cell lymphoma, high-grade B-cell lymphoma and Follicular lymphoma (FL)-transformed DLBCL based on a multicenter ZUMA-1 trial. The long-term update analysis of ZUMA-1 suggested that axicabtagene ciloleucel can induce durable responses and a median OS of greater than 2 years with 58% CR, and has a manageable long-term safety profile in patients with R/R large B-cell lymphoma (18). On March 5, 2021, axicabtagene ciloleucel was also approved for R/R FL after two or more lines of systemic therapy based on the single-arm, open-label ZUMA-5 trial. Subsequently, in June 2021, axicabtagene ciloleucel was approved by NMPA for the treatment of adult patients with R/R large B-cell lymphoma after two or more lines of systemic therapy based on results of a single-arm, open label, multi-center bridging trial (FKC876-2018-001).

Brexucabtagene autoleucel, an anti-CD19 autologous CAR-T therapy, was approved by FDA for R/R mantle cell lymphoma (MCL) on July 24, 2020 based on ZUMA-2 trial. On October 1, 2021, brexucabtagene autoleucel received another approval from the FDA to treat adults with R/R B-ALL based on results from the ZUMA-3 trial. On February 5, 2021, lisocabtagene maraleucel, an anti-CD19 autologous CAR-T therapy, was approved for the treatment of R/R large B-cell lymphoma after two or more lines of systemic therapy, which was based on the multicenter TRANSCEND trial. On September 3, 2021, relmacabtagene autoleucel, an anti-CD19 autologous CAR-T cell therapy, was approved by NMPA for the treatment of adult patients with R/R large B-cell lymphoma after two or more lines of systemic therapy which was based on the results of a multi-center RELIANCE study. On November 8, 2023, inaticabtagene Autoleucel (CNCT19), the first proprietary CD19-targeted CAR-T product invented in China, was approved by NMPA for the treatment of adult R/R B-ALL. This approval was based on the clinical results from a single-arm, multi-center, pivotal study for adult R/R B-ALL in China, in which 90% of the 20 patients treated with infusions of CNCT19 cells reached CR/CRi within 28 days, and the median OS and RFS after a median follow-up of 10.09 months were 12.91 months and 6.93 months, respectively (19).

In addition to the CD19-targeting CAR-T therapy, two CAR-T products targeting BCMA have also been approved by FDA for the treatment of R/R multiple myeloma (MM) after four or more lines of systemic therapy. The first one was the idecabtagene vicleucel, which was approved on March 26, 2021 based on the multicenter KarMMa study. In this trial, 128 patients received treatment, and the ORR was 73% (95% CI, 66 to 81) with 33% CR at a median follow-up of 13.3 months (20). Ciltacabtagene autoleucel, the second FDA-approved anti-BCMA CAR-T therapy was approved on February 28, 2022 based on the multicenter CARTITUDE-1 trial. On June 30, 2023, equecabtagene autoleucel, a fully human BCMA-targeting CAR-T cell therapy, acquired the approval of NMPA for the treatment of adult patients with R/R MM who have progressed after ≥ 3 lines of therapy containing at least a proteasome inhibitor and an immunomodulatory agent. The approval was based on data from the FUMANBA-1 study conducted in China (NCT05066646); in the first 2 weeks after infusion the ORR was 77.8% (14 of 18); at 1 month post-infusion, the ORR was 88.9% (16 of 18), with a CR or a stringent CR (sCR) rate of 44.4% (8 of 18); the ORR was 100% for all patients (18 of 18), with enhanced responses over time; and a total of 72.2% (13 of 18) of the patients finally achieved a CR or sCR; 9 of them were tested minimal residual disease (MRD) by next-generation sequencing with 4 patients achieved the best responses of MRD negativity (21). Zevorcabtagene autoleucel, another fully human BCMA-targeting CAR-T cell therapy produced by CARsgen Therapeutics, was approval by NMPA on February 23, 2024, which was based on LUMMICAR STUDY 1 (NCT03975907).

### Under clinical or preclinical studies

In addition to the authorized targets of CD19 and BCMA, other tumor-associated antigens were intensely explored under preclinical and clinical studies. The richest pipeline of new antigens was developed and targeted in B cell or Plasma cell malignancies, such as CD20, CD22, κ-light-chain, λ-light-chain, CD38, SLAMF7, GPRC5D (22–32).

CD20 and CD22 were widely expressed on the surface of mature undifferentiated B-cells or malignant B-cells, and CAR-T cells targeting either antigen have been demonstrated to be effective in specifically killing B cell leukemia and lymphoma *in vitro* and mouse models (33–35). Subsequently, the feasibility and efficacy of anti-CD20 CAR-T treatment towards R/R CD20-positive B-cell lymphoma were strongly confirmed in phase I and IIa trials. The best ORR was 9 of 11 (81.8%) with 6 patients achieving CR and 3 patients achieving PR within a median follow-up of 8 months, and no severe toxicity was observed (22). Another phase I trial demonstrated that twelve B-ALL patients (57%) achieved a CR with 9 measurable residual disease (MRD) negative after CD22 CAR-T cell infusion, including 5 patients with CD19-negative recurrence after treatment with CD19 CAR-T cells (36). Since that a single target may not completely cover all the tumor cells, dual-target CAR-T cells that allowed for either antigen to be sufficient to trigger a powerful T-cell response have been widely studied, which may contribute to prevent the relapse caused by antigen loss or modulation (37). In a phase I/II trial of tandem CD19/CD20 CAR-T therapy, the best ORR in 87 patients with R/R non-Hodgkin lymphoma (NHL) was 78% (95% CI, 68 to 86) and the median PFS was 27.6 months (38). In addition, dual-targeting of CD19 and CD22 has also achieved encouraging outcomes in clinical practice. In a pilot trial initiated by Wang and her colleagues, 89 patients with R/R B-cell malignancies received sequential infusion of anti-CD19 and anti-CD22 CAR-T. 96.0% of the 51 patients with ALL obtained MRD-negative response with median OS was 31.0 months, and 50.0% of the 38 patients with NHL achieved CR with the median OS was 18.0 months (39). In order to avoid the off-target toxicity against normal B cells, Raghuveer and other investigators have demonstrated that adoptive transfer of anti-λ CAR-T and anti-κ CAR-T cells represented a useful and alternative modality to CD19 CAR-T cells in treating B-cell malignancies with minimal impact on humoral immunity (40). In a phase I trial of κ-light-chain CAR-T cell therapy in B cell or Plasma cell malignancies, among 9 patients with relapsed NHL or chronic lymphocytic leukemia (CLL), 2 had a CR and 1 had a PR; among 7 patients with MM, 4 had stable disease lasting 2 to17 months (26). Besides, CD30 was also a promising target for classical Hodgkin lymphoma (HL), two phase I/II studies of CD30 CAR-T cells showed that the best ORR of 37 evaluable R/R HL patients was 62% with 19 patients having a CR and 4 patients having a PR (41). Furthermore, abundant clinical studies of new targets for CAR-T cell therapy in B-cell malignancies were ongoing, including CD70 (NCT05948033), Igβ (NCT05312476), ROR1 (NCT05444322, NCT05588440), BAFF (NCT05312801) and CD38 (NCT03754764).

In MM, several members of the signaling lymphocytic activation molecule (SLAM) family have been discovered as high-potential therapy targets including SLAMF7 and SLAMF3, which were uniformly expressed on chemotherapy-resistant MM cells, and CAR-T cells targeting either antigen were highly effective in preclinical experiments (30, 31, 42–44). Accordingly, clinical trials evaluating the safety and efficacy of SLAMF7 CAR-T cells were ongoing (NCT03958656, NCT03710421). A latest single-arm phase I/IIa clinical trial have investigated the feasibility and safety of bispecific CS1-BCMA CAR-T cells in patients with R/R MM, the results showed that the best ORR of 16 treated patients was 81% at a median follow-up of 246 days, and the 12-month OS and PFR were 72.73% and 56.26%, respectively (45). G protein-coupled receptor class C group 5 member D (GPRC5D), a protein normally expressed in hair follicles, was identified in MM cells and correlated with poor clinical prognosis (46). Recently, a phase I trial demonstrated the safety and efficacy of GPRC5D-targeted CAR-T cells towards MM, in which 10 (100%) of 10 patients had an overall response, including 6 (60%) stringent complete response (sCR) and 4 (40%) very good partial response (VGPR) at a median follow-up of 238 days (47). In another phase II Trial initiated by Xia, the ORR of 33 patients with R/R MM was 91% (95% CI, 76 to 98) at a median follow-up of 5.2 months, 21 patients (63%) achieving sCR or CR (48). In both two studies, all patients who received prior BCMA CAR-T cell therapy had PR or better with the GPRC5D-targeted CAR-T cells, illustrating the potential clinical utility of combinatorial therapy. Besides, given the high expression of CD38 on MM cells, targeting of CD38 (NCT05442580) or dual-targeting of CD38 and BCMA (NCT03767751) was being developed. In a phase I trial, 20 (87%) of 23 patients treating with BM38 CAR-T cells had a clinical response and MRD-negative at a median follow-up of 9.0 months (49). There were also biomarkers of MM currently under investigation as potential targets, such as CD138 (NCT01886976, NCT03672318), CD269 (NCT04500431) and LewisY (NCT01716364).

However, clinical trials of CAR-T cells in acute myeloid leukemia (AML) or T cell malignancies were limited, probably because of the paucity of ideal targets restricted to tumor cells. The broadly shared expression of targets in the hematopoietic compartment would cause severe fratricide of CAR-T cells or off-target toxicity. Nevertheless, candidate antigens of AML, such as CD7 (50), CD33 (24), CD38 (51), CD44v6 (52), CD70 (53), CD123 (25), Siglec-6 (54), CLL1 (55), FLT3 (56), FRβ (57), Le-Y (58), NKG2D ligands (59), and some towards T cell malignancies, like CD7 (60), CD5 (61), CCR9 (62), have been utilized to constructed CAR-T cells and demonstrated the efficacy in preclinical studies. CD7 was highly expressed on the surface of T-cell ALL and T-cell lymphoblastic lymphoma that making it attractive in immunotherapy. One recent research enrolled 60 patients with R/R T-cell acute lymphoblastic leukemia and lymphoblastic lymphoma (T-ALL/LBL), 58 of whom received autologous CD7 CAR-T cell therapy and 2 other patients received donor-derived CD7 CAR-T cell infusion. It was showed that 94.4% of patients achieved deep CR in bone marrow on day 28 (63). The feasibility and safety of CAR-T cells targeting other targets including CD1a (NCT05745181, NCT05679895), CD4 (NCT04162340, NCT03829540), CD5 (NCT04594135, NCT03081910), TRBC1 (NCT04828174), CD56 (NCT05941156) and CD37 (NCT04136275) were also evaluated in clinical studies for R/R T-cell leukemia/lymphoma.

In AML, a phase I study of CLL-1 CAR-T cells showed that the best ORR was 70% (7/10 patients) in adults with R/R AML at a median follow-up time of 173 days; However, CLL-1 was also expressed on granulocytes which leading to severe agranulocytosis (9/10 patients) and 2 patients died of severe infection (64). NKG2D-ligand was commonly expressed in primary AML but rarely in healthy tissues, therefore CAR-T cells targeting NKG2D-ligand has emerged as a promising treatment for R/R AML. In a phase I study of NKG2D CAR-T cells for patients with R/R AML and myelodysplastic syndromes (MDS) or MM, the best ORR of 12 evaluable patients with R/R AML or MDS was 25% at a median follow-up of 118 days, and no therapy-related deaths was observed (65). Another single-center, single-arm, phase I clinical trial (NCT03126864) also assessed the efficacy and safety of CD33 CAR-T therapy in 10 patients with R/R AML, but CAR-T cells were only successfully infused in 3 patients and they all died due to disease progression (66). Although undesirable efficacy and severe off-target toxicity were still major challenges of CAR-T therapy in R/R AML, new targets were still being investigated, such as CD123 (NCT04265963), Siglec-6 (NCT05488132), IL1RAP (NCT04169022) and FLT3 (NCT05023707). A selection of novel target antigens for CAR-T cells towards hematological malignancies that were under clinical studies were provided in **Table 1**.

**Table 1:** Clinical studies of new targets for CAR-T therapy in hematological malignancies.

Study/Ref.	Phase	Target	Number	Disease	Efficacy	Safety
ChiCTR2000036350	1	CD20	15	R/R B-NHL	The best ORR 100% with CR 80% and PR 20% at the median follow-up of 12.4 months	Gr4+ CRS 0%, NT 6.67%, Gr3+ Leukopenia 93.3%, Gr3+ Anemia 40%, Gr3+ Thrombocytopenia 60%
NCT01735604	IIa	CD20	11	R/R B-NHL	The best ORR 81.8% with CR 55% at a median follow-up of 8 months	Gr4+ CRS 0%, One suffered Gr3 herpes zoster infection
NCT02315612	1	CD22	58	R/R B-ALL	The best CR 70%, median OS 13.4 mo at a median follow-up of 24 months	Gr3+ CRS 21% , NT 37.7% (Gr2-), HLH/MAS-Like Toxicity 38%
NCT03097770	1/II	dual CD19/CD20	87	R/R B-NHL	The best ORR 78% with 70% CR and 8% PR, median PFS 27.6 mo	CRS 70%, Gr4+CRS 10%; Gr3+ICANS 2%, Two died from severe pulmonary infection
NCT00881920	1	κ-light	16	R/R B-NHL/CLL (n=9) R/R MM (n=7)	B-NHL/CLL: The best CR 22% and PR 11%; MM: The best SD 57%	One MM patient suffered Gr3 lymphopenia
NCT02690545 NCT02917083	I/II	CD30	42	R/R HL	The best ORR 62%, The best CR 51%, The best PR 10.8%, 1-year PFS 36%	CRS (Gr1) 24%, NT 0%, Gr3+ thrombocytopenia 24%, Gr3+ neutropenia 10%
NCT04689659	1	CD7	20	R/R T-ALL	CR 90% with 85% MRD- CR by day 15	AE(Gr3-4)10%, Gr3+ CRS 10%, GVHD 60%, One died from fungal pneumonia
NCT04572308	1	CD7	20	R/R T-ALL(n=14), T-LBL(n=6)	95% (19/20) CR by day 28	CRS (Gr2-) 90%, CRS (Gr3) 5%, NT (Gr1) 10%
NCT04004637	1	CD7	10	R/R T-ALL/ LBL	CR 87.5% (7/8) at 3 months after CAR T-cell infusion	CRS (Gr2-) 87.5%, NT 0%
NCT02203825	1	NKG2D	12	R/R- AML (n=7), MM (n=5)	median OS 4.7 mo, 3-mo survival rate 75%, 6-mo survival rate 42%	Gr3 AE 15%, Gr4 AE 7%
NCT03018405	1	NKG2D	12	R/R AML	ORR 25% with median follow-up of 118 days	Gr3-4 AE 44%
ChiCTR2000041054	1	CLL-1	10	R/R AML	The best CR/CRi 70% at the median follow-up time of 173 days	CRS 100%, Gr3+ CRS 60%, CRES 0%, Severe pancytopenia 100%
NCT05016778		GPRC5D	10	R/R MM	The best ORR 100% with 60% CR with a median follow-up of 238 days	CRS 100%, Gr1 CRS 90%, Gr2 CRS 10%, Hematologic toxic effects 100%
NCT04555551	1	GPRC5D	17	R/R MM	The best PR or better 71%, The best CR 35%	Gr4+ ICANS 5.9% (1/17) Gr3+ cerebellar disorder (2/17), Hematologic toxic effects
ChiCTR2100048888	II	GPRC5D	33	R/R MM	The best ORR 91% with CR 63% at a median follow-up of 5.2 months	Gr1-2 CRS 76%, NT 9%, Hematologic toxic effects, one died of intracranial hemorrhage
ChiCTR1800018143	1	BCMA CD38	23	R/R MM	The best ORR 87% with CR 52% at a median follow-up of 9.0 months, median PFS 17.2 mo	CRS 87%, Gr3+ CRS 22%, NT 0%, Hematologic toxicities, One died from pulmonary infections
NCT04662099	I/II	dual CS1/BCMA	16	R/R MM	The best ORR 81% with 38% sCR at a median follow-up of 246 days, 12-mo OS 72.73%, 12-mo PFR 56.26%	CRS 38%, NT 0%, Gr3 infection 31%

## Alternative cells used for CAR construction in hematological malignancies

In addition to the tumor-associated antigens targeted by CAR molecules, the second key element determining the anti-tumor efficacy should be the cells that carried the CARs. Currently, the FDA approved CAR-T therapies were constructed by autologous T cells derived from patients. However there has been certain well-known disadvantages, such as high costs, lengthy production process, and manufacturing failures (67, 68). Although the allogeneic CAR-T cells generated by T cells from healthy donors, so called “off-the-shelf” CAR-T cells, had superiority in manufacturing processes. The severe graft-versus-host disease (GvHD) and host immune rejection remained to be two major issues (69, 70). To overcome the above challenges, some efforts have been made including using donor-derived HLA-matched T cells, gene editing to delete TCR genes in αβ T cells and using non-αβ T cells or even non-T cells (70).

### Allogeneic HLA-matched T cells

Under the clinical application, allogeneic CAR-T cells with HLA-matched phenotype could be produced by T cells derived from an allogeneic hematopoietic stem cell transplant (allo-HSCT) donor in patients who have received an allogeneic SCT but subsequently relapsed (71). One research reported that the response of donor-derived anti-CD19 CAR-T therapy was significantly superior to that of donor lymphocyte infusion in patients with relapsed B-ALL after allo-HSCT, the best rates of MRD-negative CR were 61.5% (8/13 patients) in CAR-T treated group and 13.3% (2/15 patients) in DLI group, respectively. Only one patient developed grade 1 acute-GvHD after donor-derived anti-CD19 CAR-T cells infusion (72). Similarly, one latest study showed that the allogeneic CAR-T cells were effective in R/R children/young B-cell precursor ALL, the best CR was 100% (13/13), and 8 of 13 patients maintained CR at a median follow-up of 12 months (range, 5-21), and one patient occurred controllable acute GvHD (73). One latest research of donor-derived CD7 CAR-T cells in patients with R/R T-ALL reported that the ORR was 95% (95% CI, 76.4 to 99.1) after a median follow-up time of 27.0 months, with 85% (17/20 patients) achieved CR. The main long-term adverse events were GvHD and infection (74). Overall, these results were in line with previous reports and suggested that HLA-matched CAR-T cell therapy could be a bridge to allogeneic HSCT to reinforce the graft-versus-tumor effect without increasing the risk of GvHD (75).

### αβ TCR disrupted T cells

**Figure 2:**
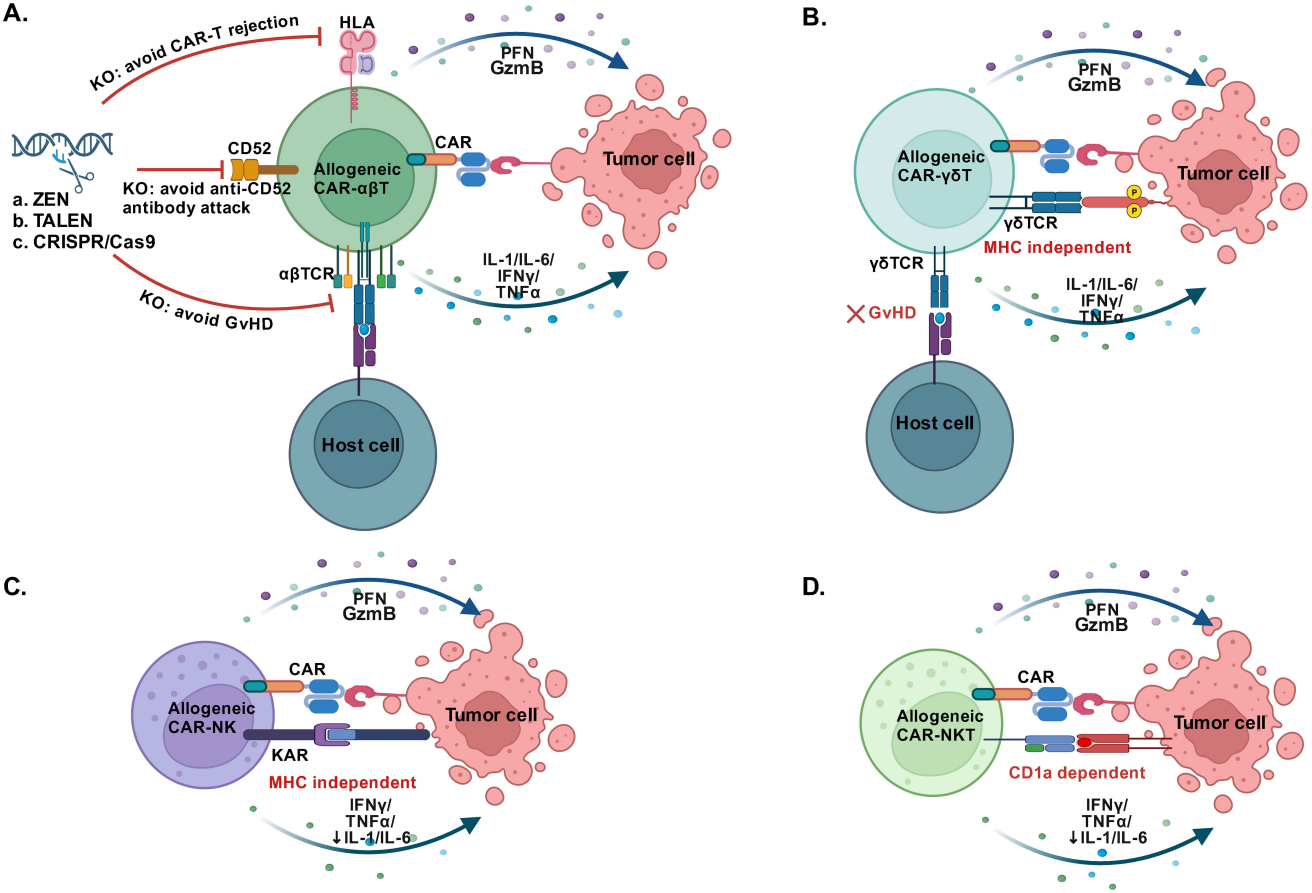
Alternative cells used for CAR construction. **(A)** CAR-T cells constructed by allogeneic αβ T cells that disrupting the αβ TCR, CD52 and HLA maintained robust antitumor efficacy, the knockout (KO) of αβ TCR relieves the GvHD, the KO of CD52 avoids the anti-CD52 antibody attack and the KO of HLA avoids CAR-T rejection. **(B)** Allogeneic CAR-γδ T cells effectively target the tumor cells in an MHC-independent manner without GvHD. **(C)** Allogeneic CAR-NK cells directly recognize tumor cells in the absence of MHC and have low risk of CRS and GvHD. **(D)** Allogeneic CAR-NKT cells recognize lipid antigens presented by MHC-like molecule CD1d and show potent antitumor activity with low risk of CRS and GvHD. GvHD, Graft-versus-host disease; PFN, Perforin; GzB, Granzyme B; KAR, Killer activatory receptors. (Created with BioRender.com).

Nevertheless, HLA-matched CAR-T cells did not meet the demand of universal allogeneic CAR-T cells that possessed scaled-up manufacturing processes and ready-to-use characteristic. Since that αβ TCR complex was the determinant of T cell alloreactivity which mediated the GvHD, preventing the expression of a functional TCR at the surface of αβ T cells may break the donor restriction. Simultaneously, the expression of HLA on allogeneic CAR-T cells also brought a CAR-T rejection effect. Studies have proved that T cells disrupting the T cell receptor constant α chain (TRAC) gene, CD52 gene and HLA gene through transcription activator-like effector nuclease (TALEN) technology maintained the antitumor properties without mediating GvHD and CAR-T rejection (69, 76)(**Figure 2A**). Clinical proof of concept was shown in two ongoing, multicentre, phase I clinical trials, in which 7 children and 14 adults with R/R B-ALL received allogeneic TALENs-edited CD19 CAR-T cells and 14 (67%) of 21 patients achieved CR/CRi 28 days after infusion. Grade 1 acute skin GvHD was occurred in 2 patients (10%) and CRS was still the most common adverse event (91%, 19/21 patients) (77). TALEN technology based allogeneic BCMA CAR-T cells were also evaluated in another dose-escalation phase I trial. Among 43 patients with R/R MM, the most frequent adverse events after lymphodepletion and allogeneic BCMA CAR-T cells infusion were neutropenia (69.8%), infection (53.5%) and CRS (55.8%), no cases of GvHD were observed; and 55.8% of patients (95% CI, 39.9 to 70.9) had a response at a median follow-up of 10.2 months (78). Moreover, allogeneic universal CAR-T cells generated utilizing CRISPR/Cas system containing two to four disrupted genes have also been developed. A phase I study investigated the feasibility of universal CD19/CD22-targeting CAR-T cells with a CRISPR/Cas9-disrupted TRAC and CD52 gene in six patients with R/R ALL. The CR rate was 83.3% (5/6 patients) on day 28 and 3 of the 5 patients who achieved CR/CRi remained MRD- negative at a median follow-up of 4.3 months. No gene editing-associated genotoxicity and GvHD were observed (79). Recently, Jaitip et al. optimized and validated a non-viral genetic modification platform based on Sleeping Beauty transposons delivered with minicircles to express CD19 CAR and CRISPR-Cas9 ribonucleoparticles to inactivate allogeneic TCR, they demonstrated these CAR-T cells possessed vigorous antitumor activities in NALM6 (B-ALL cell) tumor-bearing mice while had significantly reduced TCR alloreactivity and GvHD development (80). More clinical trials of universal allogeneic CAR-T cells were under way, these studies have consistently confirmed the efficacy and safety of universal CAR-T cells based on either ZEN, TALEN or CRISPR/Cas technology (81–83). Nevertheless, a longer follow-up and more patients enrolled in the clinical trials were required to demonstrate the efficacy and safety.

### Cord blood-derived T cells

Alternatively, the cord blood (CB)-derived T cell utilized to carry a CAR may help to meet the demand of universal allogeneic CAR-T cells due to the reduced incidence and severity of GVHD, which could be explained by the unique antigen-naive status and the impaired nuclear factor of activated T cells signaling (84). Indeed, CAR-T cells derived from CB showed a higher proportion of naive T cells and longer tumor suppression in tumor-bearing mice than those derived from patient peripheral blood (85). One clinical study was being initiated to explore the feasibility of CB-derived CAR-T cells as a treatment for B-cell malignancies (NCT03881774), and the efficacy and safety of CB-derived CAR-T cells in clinical patients deserved to be further evaluated.

### γδ T cells

Another candidate was γδ T cells, which constituted only 1-5% of circulating lymphocytes and had comparable cytotoxic effector functions and pro-inflammatory cytokine production ability to αβ T cells (86). Moreover, the unique features of γδ T cells, including MHC-independent anti-tumor activity, tissue tropism, and multivalent response against a broad spectrum of the tumors (**Figure 2B**), making them excellent candidates for designing “off‐the‐shelf” allogeneic cell products (87, 88). In a preclinical study, Meir Rozenbaum et al. successfully transduced and expanded γδ T cells with a CAR targeting CD19, and demonstrated their effective cytotoxicity against CD19-positive tumor cell lines. Besides, they also had a CAR-independent effect on CD19-negative leukemia cells (89). Another preclinical study has also proved the innate and adaptive antitumor activities of CD20 CAR-γδ T cells, and they exhibited tumor growth inhibition in B‐cell lymphoma xenografts without inducing xenogeneic GvHD. Accordingly, a phase I study has been initiated in patients with B-cell malignancies (NCT04735471) (90). In addition, a clinical trial of CAR-γδ T cells targeting CD7 was also undergoing (NCT04702841). In spite of great promise, the current challenges should be the expansion and clinically adoptable numbers of γδ T cells. And much more clinical data were needed to demonstrated the safety and efficacy of CAR γδ T cells therapy.

### Natural killer cells

Natural killer (NK) cells characterized by the expression of CD56 without co-expression of CD3 were originally identified as innate immune effector cells. Since they could directly recognize tumor cells in the absence of MHC and had low alloreactivity, allogeneic NK cells or even NK cell line hold important promise to carry a CAR for the development of “off the shelf” cancer immunotherapy (**Figure 2C**). In addition, the cytokines produced by NK cells were distinct from IL1and IL6 produced by T cells, which greatly reduced the risk of CRS and neurotoxicity (91). Recent years, studies have discovered that NK cell dysfunction occurred in different types of cancers, illustrating that tumor cells had evolved mechanisms to escape NK cell killing and reinforcing the CAR-NK cell therapy was therefore an attractive strategy (92). Currently, a large number of preclinical studies have shown great success of CAR-modified NK cells or NK-92 cell lines in various types of hematological tumors (93–95). For example, CD5 CAR-NK cells or CD7 CAR-NK cells constructed by human NK-92 cell line showed consistent, specific, and potent anti-tumor activity against a variety of T-cell leukemia and lymphoma *in vitro (* 93, 96). Initial clinical trials on CAR-NK cells have also confirmed their superiority as a treatment of hematological malignancies. Currently, Liu et al. reported a clinical trial that CB-derived HLA mismatched CD19 CAR-NK cells were successfully constructed to treat R/R CD19-positive cancers (NCT03056339). In the clinical trial, no cases of CRS, neurotoxicity and GvHD were occurred after administration of CD19 CAR-NK cells. Of the 11 patients who received CD19 CAR-NK cell treatment, 8 (73%) exhibited a response with 7 (64%) having a CR at a median follow-up of 13.8 months (range, 2.8-20.0), and 1 having remission of the Richter’s transformation component but had persistent CLL, and the infused CAR-NK cells expanded and persisted for at least 12 months (97). Huang et al. launched a phase I clinical trial of CB-derived CD33 CAR-NK cells for patients with R/R AML (NCT05008575). Among 10 evaluated patients, only one patient had grade 2 CRS and no GVHD occurred, and 6 patients (60%) achieved CR 28 days after CD33 CAR-NK cell infusion (98). The safety and tolerability of induced pluripotent stem cells (iPSCs) derived BCMA CAR-NK cells were also investigated in patients with R/R MM (NCT05182073), which was reported in the 64th ASH Annual Meeting & Exposition (99). Collectively, the combination of NK cells and CAR may become a promising cancer immunotherapy as the characteristics of HLA-independent recognition, the variety of cell sources and the reduced risk of immune effector cell-associated toxicity (100).

Furthermore, other candidate such as natural killer T (NKT) cells, iPSCs and macrophage were also under investigation. NKT cells represented a specific subset of T cells that recognized lipid antigens presented by MHC-like molecule CD1d and showed typical NK cell features (**Figure 2D**), thereby bridging the innate and adaptive immune responses (101). Preclinical trials have demonstrated the that NKT cells may possess potent antitumor activity, besides they had superiority in regulating the tumor microenvironment and reducing the risk of GVHD (102, 103). A preclinical study successfully demonstrated that CD62L-positive NKT cells transduced with a CD19-specific CAR achieved sustained tumor regression by targeting both CD19 and CD1d expressed on lymphoma cells (104). In spite of great promise for “off‐the‐shelf” CAR NKT cell product, there were limited data about the related clinical trials for patients with hematological malignancies, but CAR-NKT cells have shown satisfactory effects in patients with R/R neuroblastoma (105, 106). T cells derived from iPSCs have also been widely researched recently and considered to be a promising source for the generation of “off-the-shelf” CAR-T cells. Several studies have exhibited that CAR-T cells can be generated from reprogrammed T cell derived from hypoimmunogenic iPSCs, and proved to control tumor growth in mice model without causing GvHD (107–109). Beyond T cells and NK cells, the role of macrophages in the immunosuppressive tumor microenvironment has also attracted great interest in CAR-based therapy to enhance macrophage phagocytosis. Pluripotent stem cell-derived CD19 CAR or Meso CAR-expressing macrophage cells appeared potent antigen-dependent phagocytosis of tumor cells and *in vivo* antitumor activity (110).

All of these alternative cells have achieved promising results in improving the feasibility of CAR-based immunotherapy and expected to be approved for clinical use in the future. Of note, the safety and the efficacy of the above approaches needed to be clinically assessed. So far, abundant clinical trials with allogeneic CAR-based cells have been planned or were ongoing, and the detailed information of some clinical trials was shown at **Table 2**.

**Table 2:** Clinical studies of allogeneic CAR-based therapy in hematological malignancies.

Study	Phase	Status	Cell sources	Target	Disease	Starting time
NCT05714345	II	Recruiting	Allogeneic αβT cells with CD52 deleted	CD19	R/R LBCL	March 31, 2023
NCT05377827	I	Recruiting	Allogeneic αβT cells with TRAC deleted	CD7	R/R T-NHL R/R AML	October 10, 2023
NCT06323525	I/II	Recruiting	Allogeneic αβT cells with TRAC and Power3 double genes deleted	CD19	R/R B-NHL	April 17, 2024
NCT05722418	I	Recruiting	CRISPR-Edited allogeneic αβT cells	BCMA	R/R MM	February 10, 2023
NCT05993611	I	Recruiting	Allogeneic T regulatory cells	CD6	cGVHD	June 17, 2024
NCT05554939	I/II	Recruiting	Allogeneic γδT cells	CD19	R/R B-NHL	December 11, 2022
NCT06056752	I	Recruiting	Allogeneic γδT cells	CD19	R/R B-ALL	September 27, 2023
NCT04623944	I	not recruiting	Allogeneic NK cells	NKG2D	R/R AML R/R MDS	September 21, 2020
NCT05020678	I	Recruiting	Allogeneic NK cells	CD19	R/R B-ALL/NHL R/R CLL	August 20, 2021
NCT05379647	I	Recruiting	Allogeneic NK cells	CD19	R/R B-ALL R/R B-NHL	November 4, 2021
NCT05665075	I	Recruiting	Allogeneic NK cells	CD33	R/R AML	December 24, 2022
NCT05739227	I	Recruiting	Allogeneic NK cells	CD19	R/R B-cell malignancies	March 1, 2023
NCT05574608	I	Recruiting	Allogeneic NK cells	CD123	R/R AML	October 1, 2022
NCT02944162	I/II	Unknown	Allogeneic NK cells	CD33	R/R AML	October 2016
NCT06045091	I	Recruiting	Allogeneic NK cells	BCMA	R/R MM	July 4, 2023
NCT06325748	I	Not yet recruiting	Logic Gated allogeneic NK Cells	CD33 and/or FLT3	R/R AML R/R MDS	June 2024
NCT05487651	I	Recruiting	Allogeneic NKT cells	CD19	R/R B-cell malignancies	October 1, 2022
NCT05182073	I	Recruiting	Allogeneic iPSCs cells	BCMA	R/R MM	November 24, 2021

## Combinational strategies for ameliorating the CAR-T cell therapy

Although the combination of ideal targets and cells could effectively fight tumor cells, several challenges, such as how to improve the persistence of CAR-T cells, avoid tumor escape and the immunosuppressive effect of tumor microenvironment, still needed to be solved. Therefore, adopting combinational therapeutic strategies such as gene editing to additionally target immune checkpoints and coordinated drugs were particularly important.

**Figure 3:**
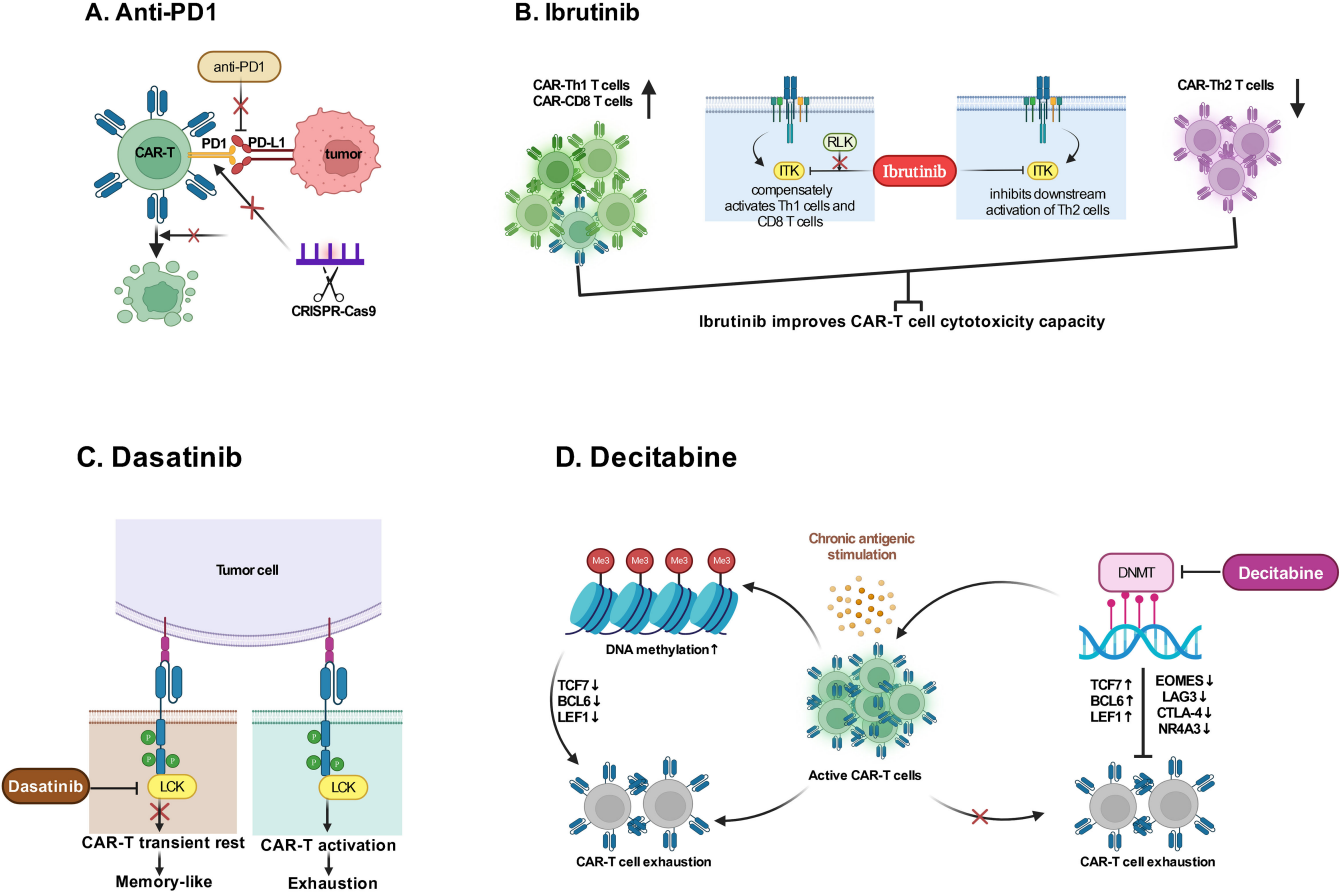
Combination therapies with drugs. **(A)** Anti-PD1 agent or editing immune checkpoint gene through CRISPR-Cas9 enhance CAR-T cytotoxicity by reducing immunosuppression in tumor microenvironment to reverse T cell exhaustion. **(B)** Ibrutinib binds ITK irreversibly to inhibit activation of Th2 cells and drive a Th1-selective pressure to maintain CAR-T cell cytotoxicity capacity. **(C)** Dasatinib promptly restrains CAR-T cell function by interfering with LCK in a way of allowing to renew CARs function after Dasatinib withdrawal to decrease T cell exhaustion. **(D)** The use of Decitabine is demonstrated to reverse T cell exhaustion due to DNA methylation caused by chronic antigenic stimulation. ITK, IL-2 inducible T-cell kinase; RLK, Receptor-like kinase; LCK, the lymphocyte-specific protein tyrosine kinase; DNMT, DNA methyltransferases; TCF7, Transcription Factor 7; BCL-6, B-cell lymphoma 6 protein; LEF, Lymphoid Enhancing Factor; EOMES, Eomesodermin; LAG3, Lymphocyte activation gene-3; CTLA-4, Cytotoxic T-lymphocyte associated protein 4; NR4A3, Nuclear Receptor Subfamily 4 Group A Member 3. (Created with BioRender.com).

Since that T-cell exhaustion and/or an immunosuppressive tumor microenvironment may result in CAR-T cell failure, the combinatorial therapy with immune checkpoint inhibitor may contribute to reverse CAR-T cell exhaustion. In a recent clinical trials (NCT02650999), 12 patients with B-cell lymphomas who were either refractory or relapsed after CD19 CAR-T cell therapy were treated with anti-PD1 agent pembrolizumab, and best ORR after pembrolizumab was 25% (3 of 12 patients; 1 CR; 2 PR); and 4 of 12 (33%) patients had clinical benefit and an increase in percentage of CAR-T cells (111). A multi-center phase II clinical trial (NCT03196830) also concluded that the combination of anti-PD-1 antibody showed an enhancement effect on CD30 CAR-T therapy in R/R CD30-positive lymphoma patients with minimal toxicities (112). Besides, the method of genetic perturbation of immune checkpoint genes has also been designed (**Figure 3A**); For example, recently a non-viral type of anti-CD19 CAR-T cell with PD1 integration through CRISPR-Cas9 has successfully developed, which showed superior ability to eradicate tumor cells in xenograft models and in adoptive therapy for eight R/R aggressive B cell NHL (NCT04213469) with a high rate (87.5%) of CR and durable responses without serious adverse events (113).

Additional combinatorial therapy not directly targeting immune checkpoints have also been utilized to strengthen the function of CAR-T cells. Researchers have observed that CLL patients who had been treated with ibrutinib for ≥1 year at the time of T-cell collection had improved ex vivo and *in vivo* expansion of CTL019, which correlated positively together with clinical response (114); and human xenograft model study has demonstrated that ibrutinib exposure did not impair CAR-T cell function *in vitro* but improved CAR-T cell engraftment, tumor clearance, and survival (114), which could be explained by a previous study (115) that ibrutinib irreversibly bound ITK and inhibits activation of Th2 cells after TCR stimulation, thus providing a compensatory platform for activation of Th1 and CD8 T cells (**Figure 3B**). A pilot study has further confirmed that CD19 CAR-T cells with concurrent ibrutinib for R/R CLL were well tolerated, with low CRS severity, and contribute to high rates (61%) of MRD-negative response (116). Preclinical studies have indicated that multi-kinase inhibitor dasatinib provided an approach to mitigate deleterious CAR signaling, which caused the transformation to a memory like phenotype and restored anti-tumor functionality in exhausted CAR-T cells (**Figure 3C**) (117, 118). Studies have also demonstrated that low-dose decitabine priming can endow CAR-T cells with enhanced and persistent anti-tumor potential via epigenetic reprogramming (**Figure 3D**) (119, 120). Furthermore, researches via integrated drug profiling or CRISPR screening and others, have identified several essential pathways for CAR-T cell cytotoxicity, which may provide a resource of immunomodulatory properties of cancer drugs and genetic mechanisms influencing CAR-T cell cytotoxicity, thus offering much more combination regimens conducive to CAR-T efficacy (121).

Another combinational strategy that can help CAR-T therapy to achieve sustained effect may be the consolidative allo-HSCT. A non-randomized interventional pragmatic clinical trial has reported that CD19 CAR-T therapy bridging to allo-HSCT for R/R B-ALL improved the long-term prognosis of the MRD-negative CR patients after CAR-T therapy; In the study, 58 R/R B-ALL patients received split doses of CAR-T cells with 51 (87.9%) achieved CR within one month, and then 21/47 MRD-negative CR patients without previous allo-HSCT and contraindications or other restrictions, on their own accord, received consolidative allo-HSCT within three months after CAR-T therapy; although there was no difference in OS between the MRD-negative CR patients who received allo-HSCT and those who did not, EFS and RFS were significantly prolonged by bridging to allo-HSCT, especially when they have high pre-infusion BM-FCM-MRD or poor prognostic markers (P<0.05) (122). In addition, another non-randomized study, in which 110 B-ALL patients received CD19 CAR-T therapy and 75 nonrandomly selected patients (73.5%) subsequently received an allo-HSCT, also exhibited that leukemia-free survival (76.9% vs 11.6%; P <.0001; 95% confidence interval [CI], 11.6-108.4) and OS (79.1% vs 32.0%; P <.0001; 95% CI, 0.02-0.22) were significantly better among patients who subsequently received allo-HSCT compared with those receiving CAR T-cell therapy alone (123). Although these two non-randomized clinical trials demonstrated that a subsequent allo-HSCT after CAR-T therapy improved eliminating leukemia cells, the benefits concerning long-term survival and quality of life still require to be further confirmed under the randomized controlled trials.

## Concluding remarks

CAR-T therapy researches have progressed rapidly into the clinic to treat hematological malignancies and now back to the bench for the reason that several significant unmet needs remained to be solved. Firstly, the high cost and the labor-intensive manufacturing process of CAR-T cells still hampered the popularization of CAR-T cell therapy; Additionally, the therapeutic efficacy of the CAR-T products, involving treatment response, disease recurrence or side effects, were still the most important issues waiting for progress; Finally, in the “real-world”, when to utilize the CAR-T therapy, and whether needing the combination therapies to ameliorate patient survival, should be answered with more evidence.

To solve the problems above, several researches appeared and proposed some data and points. Here, we outlined and updated the major advances in CAR-T eras, including the sum of current FDA-approved CAR-T drugs, researches related to novel targets for popularization of CAR-T cell therapy and alternative cell types to develop “off‐the‐shelf” CAR-T, and the combinational therapeutic strategies to improve the persistent efficiency of the CAR-T cell therapy. However, some exhilarating results were still under preclinical researches, much more clinical trials were urgently needed to further confirm the efficiency and safety; thus, we had the reasons to believe that these innovations in technology were bound to lead to improved responses and popularization of CAR-T cell therapy.
